# Compact 2-port linearly and circularly polarized antennas using low cross-polarization miniaturized patch

**DOI:** 10.1038/s41598-026-46704-6

**Published:** 2026-04-02

**Authors:** Anh Tran-Tuan, Trang Hoang-Thu, Tu Le-Tuan, Mohammad Alibakhshikenari, Yazeed Mohammad Qasaymeh, Takfarinas Saber, Patrizia Livreri

**Affiliations:** 1 Faculty of Electrical and Electronic Engineering, PHENIKAA School of Engineering, PHENIKAA University, Hanoi, 12116 Vietnam; 2 LERO, the Research Ireland Centre for Software, College of Science and Engineering, School of Computer Science, University of Galway, Galway, H91 TK33 Ireland; 3 Department of Electrical and Electronics Engineering, Dogus University, 34775 Umraniye, Istanbul Türkiye; 4 Department of Electrical Engineering, College of Engineering, Majmaah University, 11952 Al-Majmaah, Saudi Arabia; 5 Department of Engineering, University of Palermo, 90128 Palermo, PA Italy

**Keywords:** Engineering, Physics

## Abstract

This paper presents compact two-port antenna arrays with linear and circular polarization based on a miniaturized patch radiator with extremely low cross-polarization radiation. The primary radiator employs a conventional rectangular patch loaded with multiple slots and meander-line structures to increase the equivalent capacitance, thereby lowering the resonant frequency without enlarging the antenna footprint. Owing to the inherently low cross-polarization radiation of the miniaturized radiator, two compact 2-port array configurations are implemented: the first provides polarization diversity under linear polarization, while the second employs a hybrid coupler to achieve dual circular polarization. Both arrays operate at 4.3 GHz and possess compact physical dimensions with stable radiation performance. Compared with state-of-the-art works, the proposed designs exhibit a significantly reduced size while maintaining competitive radiation characteristics, making them suitable for space-constrained wireless systems.

## Introduction

Two-port antennas with dual linear or dual circular polarization are vital for modern wireless systems because they provide polarization diversity that enhances link reliability, mitigates multipath fading, and improves MIMO channel capacity. Achieving high port isolation in a compact structure remains challenging, motivating continued research on efficient dual-polarized antenna designs^1,2^. Various two-port antennas with linear polarization have been reported in the literature, where two closely spaced patch elements are electromagnetically decoupled using different isolation techniques. Near-field resonators (NFRs)^3,4^, metasurfaces^5,6^, dielectric blocks^7^, and dielectric walls^8^ have been demonstrated to effectively suppress mutual coupling. However, these decoupling structures are typically implemented above or between the radiating elements, resulting in a considerable increase in the antenna dimensions in both vertical and horizontal directions.

To improve compactness, placing the decoupling structures in the same layer as the radiators has been explored. In this regard, the two-port antennas reported in^9,10^ employ polarization conversion techniques, while electromagnetic band-gap (EBG) structures are utilized in^11–14^. Alternatively, grounded stubs and defected ground structures (DGSs) have been introduced in^15–18^. Although high isolation can be achieved, these designs generally require large physical dimensions due to the inherent half-wavelength patch size and the additional space occupied by the decoupling networks. Consequently, the use of quarter-wavelength patches^19–21^ or self-decoupling approaches^22–24^ has been proposed to enhance compactness; nevertheless, large antenna size remains a notable limitation.

For circularly polarized (CP) operation, several MIMO antennas employing CP radiation have been reported in the literature. In^25–28^, two-element CP MIMO antennas exhibit relatively narrow impedance and axial-ratio bandwidths (BWs), where defected ground structures (DGSs) are employed to enhance isolation. In contrast, the incorporation of metasurfaces (MSs) or parasitic elements (PEs) in^29,30^ has been shown to simultaneously improve mutual coupling suppression and broaden the operating BW. Nevertheless, these approaches generally result in increased antenna size and structural complexity, limiting their suitability for compact and low-profile MIMO platforms.

This paper presents two different compact two-port antennas based on a miniaturized patch radiator. The proposed design utilizes a conventional rectangular patch loaded with multiple slots and meander-line structures to effectively increase the equivalent capacitance, thereby reducing the resonant frequency without enlarging the physical footprint. Benefiting from the inherently low cross-polarization radiation of the miniaturized patch, two compact two-port array configurations are realized. The first configuration provides polarization diversity with linear polarization (LP), while the second employs a hybrid coupler to achieve dual-CP realization.

## Effect of cross polarization on the 2-port antenna system

Low cross-polarization is a critical requirement when designing 2-port antenna systems, especially in MIMO, diversity, and dual-polarized configurations. When each port transmits or receives a specific polarization state, unwanted cross-polarized radiation introduces polarization impurity and lowers channel isolation. A low cross-polarization element ensures that each port maintains high polarization purity, enabling better signal discrimination and reducing interference between ports. This is especially important in compact platforms where spatial separation is limited, and polarization orthogonality becomes one of the few effective mechanisms to enhance isolation.

Coupling between two LP antennas placed in proximity is governed by mutual impedance. When antennas are closely spaced, the electromagnetic near fields strongly interact, causing a portion of the power from one antenna to be transferred into the other, which deteriorates isolation and increases correlation—undesirable effects in multi-antenna systems. LP antennas aligned parallel have the highest coupling due to strong co-polarized field overlap, while orthogonal orientation reduces coupling through polarization mismatch.

**Fig. 1:**
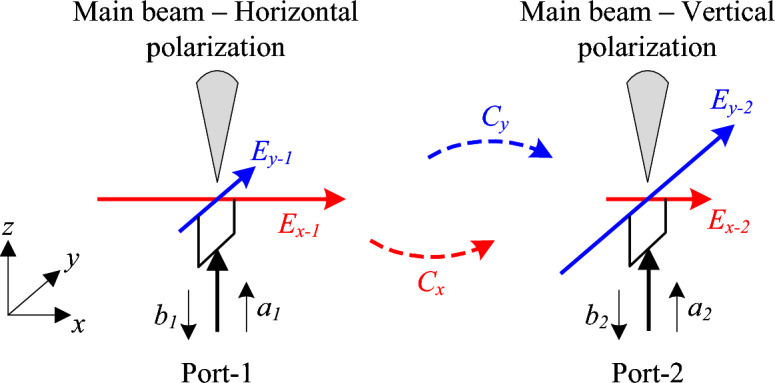
Coupling behavior between two LP antennas.

Figure 1 shows the coupling behavior between two antennas with orthogonal polarization. In practical antenna systems, port-to-port coupling can be influenced by several additional factors, including near-field interactions, current distributions, radiator geometry, and feeding structures. A rigorous analytical model that incorporates all these effects would significantly increase the complexity of the analysis and is beyond the scope of this conceptual explanation. Therefore, to maintain clarity, the discussion focuses on the most important factor, namely the interaction between the electric-field components. Antenna-1 with Port-1 excitation radiates co-polarization with E-field in x-direction ($E_{x-1}$) and cross-polarization with E-field in y-direction ($E_{y-1}$). In contrast, Antenna-2 with Port-2 operation radiates co-polarization in orthogonal direction ($E_{y-2}$) and cross-polarization ($E_{x-2}$). In this figure, ($a_1$, $b_1$) and ($a_2$, $b_2$) denote the input and output power of Port-1 and Port-2. Theoretically, the coupling between two fields is denoted as *C*, which is proportional to the dot product of their polarization vector, $C \propto |\textbf{E}_1 \cdot \textbf{E}_2|$. $C_x$ and $C_y$ are the coupling caused by the E-fields in x- and y-direction. If $\textbf{E}_1$ and $\textbf{E}_2$ are orthogonal fields, the coupling will be equal to zero due to $\textbf{E}_1 \cdot \textbf{E}_2 = 0$. On the other hand, the coupling will be maximized when the E-fields are in the same direction, $\textbf{E}_1 \cdot \textbf{E}_2 = 1$.

Let’s see the circumstance shown in Fig. 1. When Port-1 is excited, the coupling to Port-2 is denoted as $S_{21} = \frac{b_2}{a_1}\big |_{a_2=0}$. The power flowing to Port-2, $b_2$, is caused by the coupling from $E_{x-1}$ and $E_{y-1}$ to $E_{x-2}$ and $E_{y-2}$. Here, $b_2$ can be quantitatively defined by the following equation:

1 $$\begin{aligned} b_2= & C_x\left( \left| E_{x-1} \cdot E_{x-2}\right| +\left| E_{x-1} \cdot E_{y-2}\right| \right) +C_y\left( \left| E_{y-1} \cdot E_{x-2}\right| +\left| E_{y-1} \cdot E_{y-2}\right| \right) \nonumber \\ & =C_x\left( \left| E_{x-1} \cdot E_{x-2}\right| \right) +C_y\left( \left| E_{y-1} \cdot E_{y-2}\right| \right) \end{aligned}$$

As $E_{x-1}$ is orthogonal to $E_{y-2}$, and $E_{y-1}$ is orthogonal to $E_{x-2}$, the coupling between these fields is approximately zero. According to the above equation, $b_2$ will be small when two cross-polarization fields of two antennas ($E_{y-1}$ and $E_{x-2}$) are small.

To design compact 2-port antennas, a compact radiator is required. However, the compact radiator inherently suffers from increased cross-polarization due to structural asymmetry, surface-wave excitation, and strong fringing fields. When the radiator size is significantly reduced, current paths become distorted and non-uniform, leading to undesired fields orthogonal to the intended polarization. Therefore, achieving low cross-polarization in compact patches remains a design challenge because size miniaturization typically compromises radiation purity, efficiency, and BW. It is also noted that the quarter-wavelength patch can achieve the compact size, but extremely high cross polarization (-10 dB) due to the asymmetrical geometry. This paper will propose a miniaturized patch with extremely low cross polarization, while having the similar size as the conventional quarter-wavelength patch. The proposed design is utilized to design compact 2-port antenna systems, which is the most contribution of the proposed work.

## Design a compact low cross-polarization radiator

**Fig. 2:**
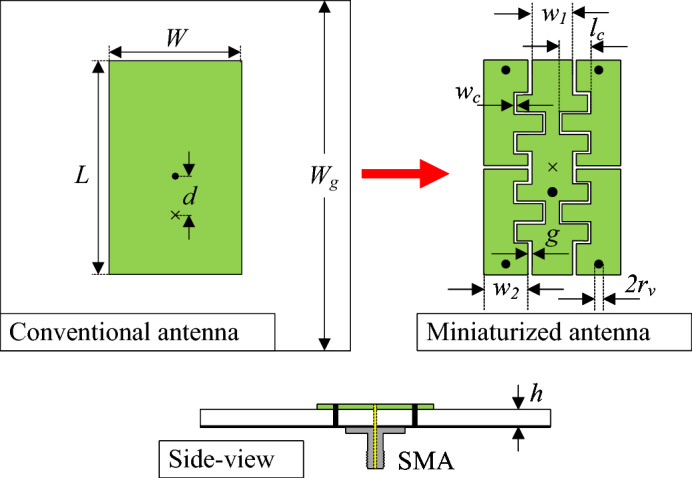
Geometry of the conventional and proposed miniaturized antennas with the same dimensions (L, W).

Figure 2 depicts the physical layout of the proposed miniaturized patch antenna, which is fabricated on a Taconic RF-35 substrate with a relative permittivity of 3.5 and a thickness of 1.5 mm. Antenna miniaturization is realized by partitioning the patch into multiple electrically coupled sections and introducing several metallic vias to connect selected segments to the ground plane. This structural modification perturbs the current paths and introduces strong capacitive coupling across the narrow gaps between adjacent sections. In addition, a meander-line configuration is embedded into the radiating patch to further extend the effective electrical length. Through these combined techniques, significant size reduction is achieved. The optimal patch dimensions are $L = 9.8$, $W = 6.4$, $g = 0.2$, $d = 1.0$, $l_c = 1.3$, $w_c = 0.1$, $w_1 = 1.8$, $w_2 = 2.0$, $s = 2$, $r_v = 0.2$ (unit: mm).

**Fig. 3:**
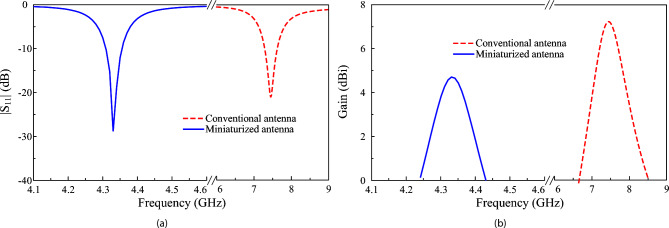
Simulated (**a**) $|S_{11}|$ and (**b**) broadside gain results of different antennas.

Figure 3 illustrates the simulated reflection coefficient and realized gain results of the conventional and proposed miniaturized patch antennas. The conventional design operating at the fundamental mode resonates at 7.5 GHz and achieves broadside gain of about 7.5 dBi. Meanwhile, the miniaturized antenna resonates at lower frequency range. A simulated -10 dB impedance BW is about 50 MHz (4.31–4.36 GHz). Besides, the broadside realized gain remains above 4.0 dBi throughout the operating range, with a maximum value of 4.7 dBi obtained at 4.33 GHz.

**Fig. 4:**
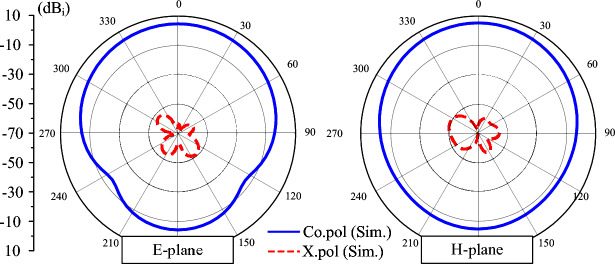
Simulated radiation patterns at 4.33 GHz of the miniaturized patch.

The simulated E-plane and H-plane radiation patterns of the proposed antenna at 4.33 GHz are presented in Fig. 4. Both co-polarized and cross-polarized components are evaluated. The results indicate symmetric radiation characteristics with respect to the broadside direction, confirming stable directional behavior. Additionally, low cross-polarization levels of smaller than -50 dB are observed, demonstrating good polarization purity.

**Fig. 5:**
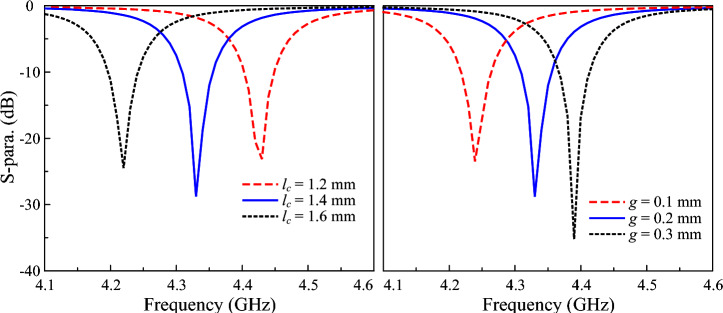
Simulated $|S_{11}|$ for different values of $l_c$ and *g*.

The gap load plays an important role in determining the operating frequency of the proposed miniaturized antenna. Figure 5 shows the simulated $|S_{11}|$ against the variations of $l_c$ and *g*. As seen, longer $l_c$ or smaller *g* results in higher capacitance. Accordingly, the resonance frequency occurs at lower frequency band.

## Compact 2-port linearly polarized antenna

**Fig. 6:**
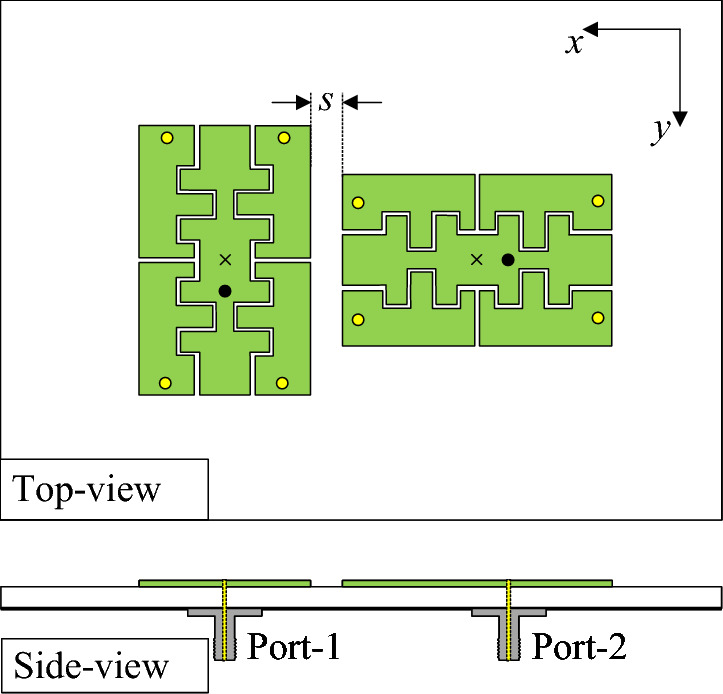
Geometry of the proposed 2-port dual-LP antenna.

Figure 6 shows the geometrical configuration of the 2-port antenna using the proposed miniaturized patches. They are positioned orthogonally with an edge-to-edge distance of 0.028$\lambda$ at the desired frequency. The optimal design dimensions are $L_s = 30$, $W_s = 30$, $h = 1.52$, $L = 9.8$, $g = 0.2$, $d = 1.0$, $l_c = 1.3$, $w_c = 0.1$, $w_1 = 1.8$, $w_2 = 2.0$, $s = 2$, $r_v = 0.2$ (unit: mm).

**Fig. 7:**
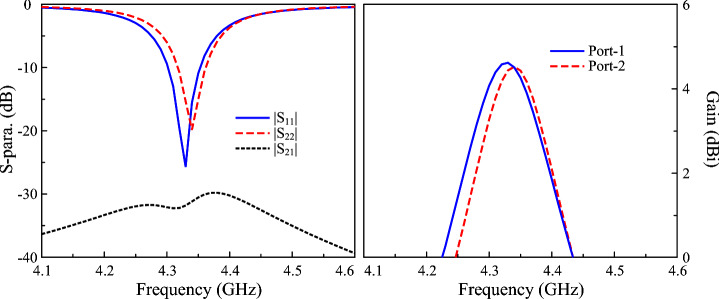
Simulated S-parameter and gain of the proposed 2-port MIMO antenna.

Figure 7 illustrates the simulated S-parameters and gain of the proposed two-port antenna. Both ports exhibit closely matched input impedance characteristics, with $|S_{11}|$ and $|S_{22}|$ remaining below -10 dB over the operating frequency range of 4.32–4.36 GHz, indicating satisfactory impedance matching. The mutual coupling, quantified by $|S_{21}|$, is suppressed to levels better than -30 dB across the same band, confirming effective electromagnetic decoupling between the radiating elements. The corresponding simulated broadside realized gain demonstrating values consistently exceeding 3.8 dBi throughout the passband and reaching a maximum of approximately 4.6 dBi.

**Fig. 8:**
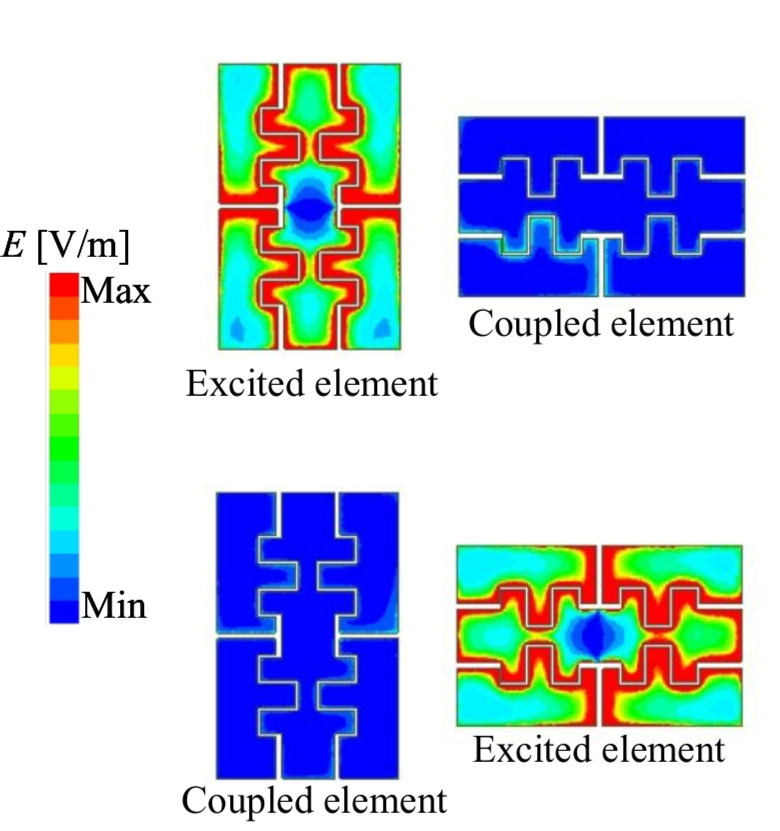
Simulated E-field distribution at 4.34 GHz.

Figure 8 shows the simulated near-field electric-field distribution at 4.34 GHz with different excitations. The strong field localization around the driven element and negligible field intensity on the inactive port provide physical insight into the achieved high isolation, as the coupling path is effectively suppressed by the proposed decoupling configuration.

## Compact 2-port circularly polarized antenna

**Fig. 9:**
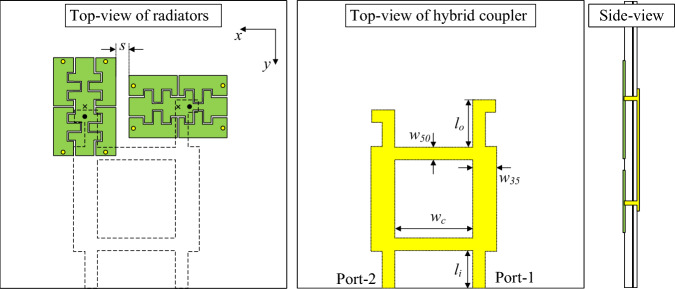
Geometry of the proposed 2-port dual-CP antenna.

Figure 9 shows the geometrical configuration of the 2-port antenna with dual-CP realization. They are positioned orthogonally with an edge-to-edge distance of 1.3 mm, corresponding to about 0.018$\lambda$ at the desired frequency. For dual-CP realization, the hybrid coupler is used, and it is printed on the FR-4 substrate with dielectric constant of 4.4. The optimal design dimensions are $L_s = 40$, $W_s = 30$, $h = 1.52$, $L = 9.8$, $g = 0.2$, $d = 1.0$, $l_c = 1.3$, $w_c = 0.1$, $w_1 = 1.8$, $w_2 = 2.0$, $s = 2$, $r_v = 0.2$, $w_c = 7.8$, $w_{35} = 2.4$, $w_{50} = 1.3$, $l_i = 3.7$, $l_o = 4.7$ (unit: mm).

**Fig. 10:**
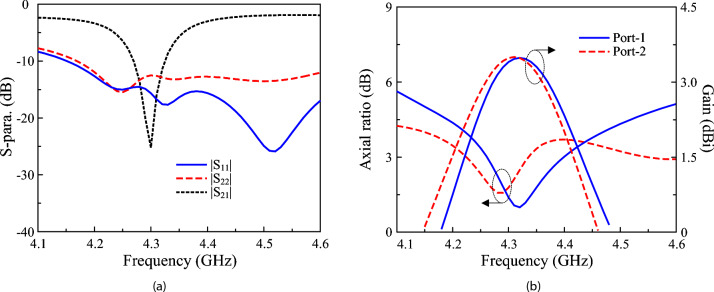
Simulated (**a**) S-parameter and (**b**) AR and gain of the 2-port dual-CP antenna.

The simulated S-parameters, AR, and gain characteristics of the proposed 2-port dual-CP antenna are presented in Fig. 10. The antenna exhibits an operating band from 4.27 to 4.33 GHz, centered at 4.3 GHz. Within this band, both ports achieve reflection coefficients below -10 dB, while the inter-port isolation remains higher than 10 dB, with a peak isolation of approximately 25 dB at 4.3 GHz. The axial ratio stays below 3 dB throughout the operating band, confirming stable circular polarization performance. The realized gain varies from 2.95 to 3.5 dBiC across the operating band.

**Fig. 11:**
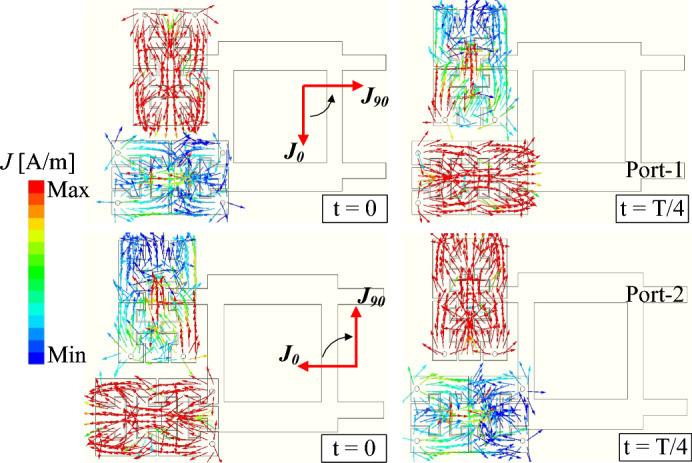
Simulated current distribution of the 2-port dual-CP antenna.

To validate the circular polarization performance, the simulated surface current distributions at 0$^{\circ }$ and 90$^{\circ }$ phase intervals are illustrated in Fig. 11. When Port-1 is fed, the surface currents exhibit a counterclockwise rotation, confirming right-hand circular polarization (RHCP). Conversely, excitation at Port-2 produces a clockwise current rotation, indicative of left-hand circular polarization (LHCP).

## Measurement results

### 2-port dual-LP antenna

**Fig. 12:**
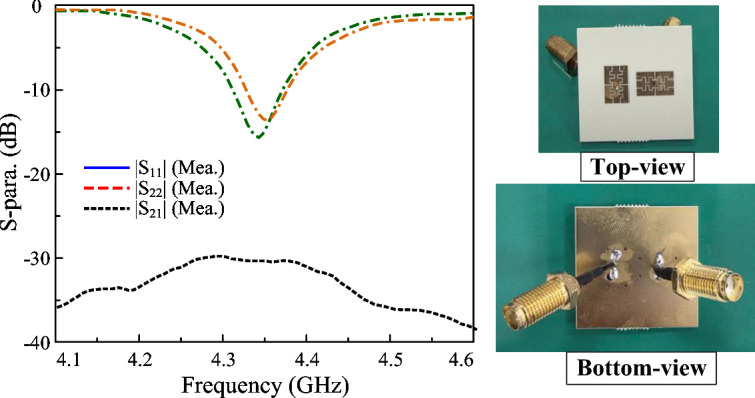
Measured S-parameter of the proposed 2-port dual-LP antenna.

**Fig. 13:**
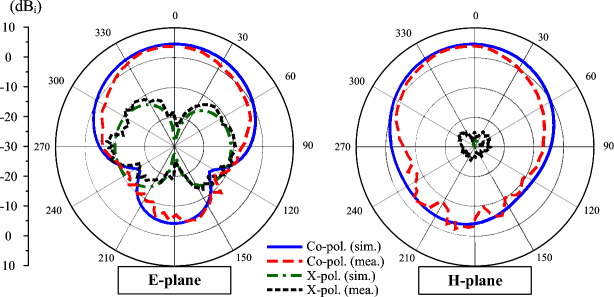
Simulated and measured gain radiation patterns of the proposed 2-port dual-LP antenna.

The measured S-parameter of the proposed 2-port dual-LP antenna is presented in Fig. 12. As seen, the operating BW with good matching performance and isolation of better than 10 dB ranges from 4.33 to 4.37 GHz. The simulated and measured radiation patterns with Port-1 operation are plotted in Fig. 13. The results for Port-2 are quite similar and thus, they are not shown for brevity. In E- and H-plane, the antenna radiates symmetrical radiation pattern with low cross-polarization radiation. The measured gain in the broadside direction is about 4.0 dBi. The polarization isolation defined by the difference between the co- and cross-polarization in the main direction is about 24 dB.

### 2-port dual-CP antenna

**Fig. 14:**
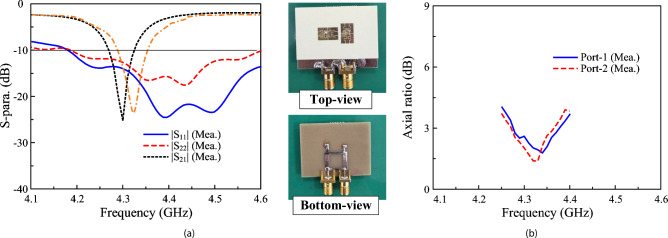
Measured (**a**) S-parameter and (**b**) AR of the proposed 2-port dual-CP antenna.

**Fig. 15:**
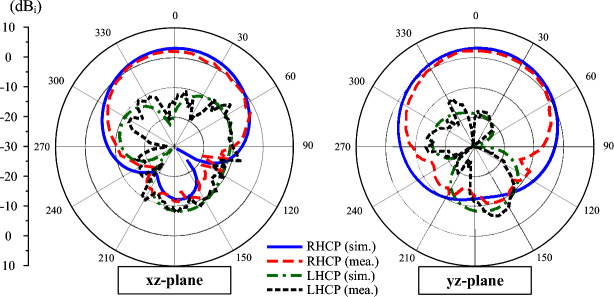
Simulated and measured gain radiation patterns of the proposed 2-port dual-CP antenna.

The measured S-parameter of the proposed 2-port dual-CP antenna is presented in Fig. 14. As seen, the operating BW with good matching and AR performance and isolation of better than 10 dB ranges from 4.29 to 4.35 GHz. The simulated and measured radiation patterns when Port-1 is excited are plotted in Fig. 15. The results for Port-2 are quite similar and thus, they are not shown for brevity. In E- and H-plane, the antenna radiates symmetrical radiation pattern with low cross-polarization radiation. The measured broadside gain is about 2.9 dBiC. Meanwhile, the polarization isolation defined by the difference between the co- and cross-polarization in the main direction is about 18 dB.

## Performance comparison

To demonstrate the compactness of the proposed antennas, Tables 1 and 2 make a comparison among the related works. Here, the radiator size is determined by the total occupied area of two radiating elements and they are defined in terms of free-space wavelength at the center operating frequency. As summarized, the proposed antennas are one of the most compact footprints among reported 2-port LP and CP antennas, while eliminating the need for decoupling networks. Despite its ultra-small electrical size, high port isolation is maintained through intrinsic modal orthogonality, reaching $\ge$ 30 dB for LP operation and $\ge$ 10 dB for CP operation. Although the achievable BW and gain are moderate, they remain competitive with other highly miniaturized designs and are obtained without sacrificing antenna simplicity or footprint. These characteristics indicate that the proposed antenna provides an excellent compromise between compactness, isolation, and radiation performance, making it well suited for space-constrained wireless applications.

**Table 1:** Performance comparison among compact 2-port LP antenna.

Ref.	Radiator size ($\lambda$)	Decoupling network	BW (%)	Isolation (dB)	Gain (dBi)
^11^	$0.74 \times 0.45 \times 0.02$	EBG	2.5	$\ge$ 35	5.0
^12^	$0.66 \times 0.26 \times 0.02$	EBG	3.3	$\ge$ 24	4.6
^13^	$0.54 \times 0.50 \times 0.02$	EBG	4.1	$\ge$ 25	4.2
^15^	$0.62 \times 0.25 \times 0.03$	Grounded stub	5.0	$\ge$ 25	5.0
^17^	$0.61 \times 0.23 \times 0.02$	T-shaped stub	5.8	$\ge$ 15	N/A
^20^	$0.29 \times 0.14 \times 0.02$	DGS	1.2	$\ge$ 30	3.8
^21^	$0.30 \times 0.11 \times 0.02$	DGS	1.4	$\ge$ 24	2.6
^23^	$0.82 \times 0.32 \times 0.04$	No	3.1	$\ge$ 30	7.0
^24^	$0.78 \times 0.25 \times 0.02$	No	1.7	$\ge$ 20	5.0
Prop.	$0.26 \times 0.14 \times 0.02$	No	1.0	$\ge$ 30	4.0

**Table 2:** Performance comparison among compact 2-port CP antenna.

Ref.	Radiator size ($\lambda$)	Decoupling network	BW (%)	Isolation (dB)	Gain (dBi)
^25^	$0.57 \times 0.27 \times 0.01$	DGS	1.9	$\ge$ 10	6.1
^26^	$0.81 \times 0.30 \times 0.02$	DGS	N/A	$\ge$ 30	6.3
^27^	$0.98 \times 0.32 \times 0.03$	Parasites + DGS	2.1	$\ge$ 20	7.7
^28^	$0.44 \times 0.21 \times 0.03$	Grounded stub	2.1	$\ge$ 20	4.5
^29^	$0.62 \times 0.55 \times 0.03$	Grounded stub	8.3	$\ge$ 26	6.2
^30^	$1.84 \times 0.92 \times 0.05$	No	16.8	$\ge$ 10	11
Prop.	$0.25 \times 0.14 \times 0.03$	No	1.4	$\ge$ 10	2.9

## Conclusion

This paper presented compact two-port antenna arrays with LP and CP radiation based on a miniaturized patch radiator. By loading the patch with multiple slots and meander-line structures, the resonant frequency is effectively lowered without increasing the antenna footprint. Two 2-port antenna configurations were implemented, providing polarization diversity under dual-LP and dual-CP using the hybrid coupler. The proposed antennas exhibit stable radiation performance within an ultra-compact size around 4.3 GHz. In comparison with existing designs, the proposed antennas offer a substantially reduced footprint while preserving comparable radiation performance, rendering them suitable for space-limited wireless applications.

## Data Availability

Data is provided within the manuscript.

## References

[1] Wong, K.-L. *Compact and Broadband Microstrip Antennas* (Wiley, New York, 2004).

[2] Elalaouy, O., Ghzaoui, E. L. M. & Foshi, J. A high-isolated wideband two-port mimo antenna for 5g millimeter-wave applications. *Results Eng.* **23**, 102466. 10.1016/j.rineng.2024.102466 (2024).

[3] Luo, S., Zhang, Y., Mei, P., Pedersen, G. F. & Zhang, S. Decoupling for millimeter-wave array antennas using near-field shrinking dielectric superstrate. *IEEE Open J. Antennas Propag.* **4**, 1187–1194. 10.1109/OJAP.2023.3328813 (2023).

[4] Singh, M., Tomar, P. S. & Parihar, M. S. A highly isolated mimo antenna system using near-field suppression mechanisms for sub-6 GHz band applications. *IETE J. Res.* **71**, 1244–1253. 10.1080/03772063.2024.2448587 (2025).

[5] Zou, X., Wang, G., Wang, Y. & Zong, B. Metasurface-based coupling suppression for wideband multiple-input-multiple-output antenna arrays. *Opt. Express* **29**, 41643. 10.1364/oe.444293 (2021).

[6] Wang, Z., Li, C., Wu, Q. & Yin, Y. A metasurface-based low-profile array decoupling technology to enhance isolation in mimo antenna systems. *IEEE Access* **8**, 125565–125575. 10.1109/ACCESS.2020.3007188 (2020).

[7] Yang, C., Lu, K. & Leung, K. W. Dielectric decoupler for compact mimo antenna systems. *IEEE Trans. Antennas Propag.* **70**, 6444–6454. 10.1109/tap.2022.3177555 (2022).

[8] Fang, Y., Tang, M. & Zhang, Y. P. A decoupling structure for mutual coupling suppression in stacked microstrip patch antenna array. *IEEE Antennas Wireless Propag. Lett.* **21**, 1110–1114. 10.1109/LAWP.2022.3158420 (2022).

[9] Cheng, Y.-F., Ding, X., Shao, W. & Wang, B.-Z. Reduction of mutual coupling between patch antennas using a polarization-conversion isolator. *IEEE Antennas Wireless Propag. Lett.* **16**, 1257–1260. 10.1109/LAWP.2016.2631621 (2017).

[10] Singh, G., Kumar, S., Kanaujia, B. K. & Pandey, V. K. Design and implementation of a compact tri-band four-port multiple-input-multiple-output antenna. *Int. J. RF Microwave Comput.-Aided Eng.*10.1002/mmce.23218 (2022).

[11] Yang, X., Liu, Y., Xu, Y.-X. & Gong, S.-X. Isolation enhancement in patch antenna array with fractal uc-ebg structure and cross slot. *IEEE Antennas Wireless Propag. Lett.* **16**, 2175–2178. 10.1109/LAWP.2017.2703170 (2017).

[12] Sharma, K. & Pandey, G. P. Two port compact mimo antenna for ism band applications. *Progress Electromagn. Res. C* **100**, 173–185. 10.2528/pierc20011504 (2020).

[13] Sanmugasundaram, R., Natarajan, S. & Rajkumar, R. A compact mimo antenna with electromagnetic bandgap structure for isolation enhancement. *Progress Electromagn. Res. C* **107**, 233–244. 10.2528/pierc20111306 (2021).

[14] Babu, N. S., Ansari, A. Q., Kanaujia, B. K., Singh, G. & Kumar, S. A two-port UWB MIMO antenna with an EBG structure for WLAN/ISM applications. *Mater. Today Proc.* **74**, 334–339. 10.1016/j.matpr.2022.08.316 (2023).

[15] Tran, H.-H., Nguyen, T.T.-L. & Nguyen Thi, T. Two closely spaced microstrip patches with high isolation for full-duplex/mimo applications. *PLoS ONE* **18**, e0290980. 10.1371/journal.pone.0290980 (2023). 37812615 10.1371/journal.pone.0290980 PMC10561854

[16] Dash, J. C. & Sarkar, D. Microstrip patch antenna system with enhanced inter-port isolation for full-duplex/mimo applications. *IEEE Access* **9**, 156222–156228. 10.1109/ACCESS.2021.3128997 (2021).

[17] Ghannad, A. A., Khalily, M., Xiao, P., Tafazolli, R. & Kishk, A. A. Enhanced matching and vialess decoupling of nearby patch antennas for mimo system. *IEEE Antennas Wireless Propag. Lett.* **18**, 1066–1070. 10.1109/LAWP.2019.2906308 (2019).

[18] Hwangbo, S., Yang, H. Y. & Yoon, Y.-K. Mutual coupling reduction using micromachined complementary meander-line slots for a patch array antenna. *IEEE Antennas Wireless Propag. Lett.* **16**, 1667–1670. 10.1109/LAWP.2017.2663114 (2017).

[19] Kurup, H. B., Remsha, M., Antony, D. & Rodrigues, S. Development and analysis of two quarter wavelength patch antennas. *ECS Trans.* **107**, 2495–2502. 10.1149/10701.2495ecst (2022).

[20] Kim-Thi, P. & Pham-Danh, T. Compact and high isolated microstrip patch antenna system for full-duplex/mimo applications. *Heliyon* **10**, e38980. 10.1016/j.heliyon.2024.e38980 (2024). 39435113 10.1016/j.heliyon.2024.e38980 PMC11492582

[21] Hoang-Thi, T., Tran, N., Dinh Nguyen, T. & Tran-Huy, H. Compact microstrip patch antenna array for mimo IoT applications. *Phys. Scr.* **100**, 105542. 10.1088/1402-4896/ae116b (2025).

[22] Lai, Q. X., Pan, Y. M. & Zheng, S. Y. A self-decoupling method for mimo antenna array using characteristic mode of ground plane. *IEEE Trans. Antennas Propag.* **71**, 2126–2135. 10.1109/TAP.2023.3240561 (2023).

[23] Lin, H. et al. Weak-field-based self-decoupling patch antennas. *IEEE Trans. Antennas Propag.* **68**, 4208–4217. 10.1109/TAP.2020.2970109 (2020).

[24] Kim-Thi, P., Van, T. N. & Thanh, T. B. A self-decoupling technique for isolation enhancement in closely-spaced mimo patch antennas. *IEEE Antennas Wireless Propag. Lett.* **23**, 1695–1699. 10.1109/lawp.2024.3367036 (2024).

[25] Jamal, M. Y., Li, M. & Yeung, K. L. Isolation enhancement of closely packed dual circularly polarized mimo antenna using hybrid technique. *IEEE Access* **8**, 11241–11247. 10.1109/ACCESS.2020.2964902 (2020).

[26] Gao, D., Cao, Z.-X., Fu, S.-D., Quan, X. & Chen, P. A novel slot-array defected ground structure for decoupling microstrip antenna array. *IEEE Trans. Antennas Propag.* **68**, 7027–7038. 10.1109/TAP.2020.2992881 (2020).

[27] Sufian, M. A. et al. Mutual coupling reduction of a circularly polarized mimo antenna using parasitic elements and dgs for v2x communications. *IEEE Access* **10**, 56388–56400. 10.1109/ACCESS.2022.3177886 (2022).

[28] Tran, H.-H., Nguyen, T.T.-L., Ta, H.-N. & Pham, D.-P. Coupling reduction of extremely closely spaced circularly polarized mimo patch antenna by phase shift method. *IEEE Access* **11**, 65347–65353. 10.1109/ACCESS.2023.3289840 (2023).

[29] Tran, H.-H., Hussain, N., Park, H. & Nguyen-Trong, N. Isolation in dual-sense cp mimo antennas and role of decoupling structures. *IEEE Antennas Wireless Propag. Lett.* **21**, 1203–1207. 10.1109/lawp.2022.3161669 (2022).

[30] Hussain, N., Jeong, M.-J., Abbas, A. & Kim, N. Metasurface-based single-layer wideband circularly polarized mimo antenna for 5g millimeter-wave systems. *IEEE Access* **8**, 130293–130304. 10.1109/access.2020.3009380 (2020).

